# Relationship between Treatment Burden, Health Literacy, and Medication Adherence in Older Adults Coping with Multiple Chronic Conditions

**DOI:** 10.3390/medicina59081401

**Published:** 2023-07-31

**Authors:** Dharrshinee Selvakumar, Palanisamy Sivanandy, Pravinkumar Vishwanath Ingle, Kumutha Theivasigamani

**Affiliations:** 1 School of Postgraduate Studies, International Medical University, Kuala Lumpur 57000, Malaysia; 2 Department of Pharmacy Practice, School of Pharmacy, International Medical University, Kuala Lumpur 57000, Malaysia; 3 Department of Pharmacy Practice, Nanda College of Pharmacy, Erode 638052, India

**Keywords:** diabetes, hypertension, hyperlipidaemia, health, elderly, complications

## Abstract

A prospective study was conducted to investigate the impact of treatment burden and health literacy on medication adherence among older adults with multiple chronic conditions (MCC) and to explore the potential moderating effects of demographic and clinical factors. Face-to-face structured interviews were conducted among older adults aged 60 and above using the Burden of Treatment Questionnaire (TBQ-15), Short Form Health Literacy Questionnaire (HLS-SF12), and Malaysia Medication Adherence Assessment Tool (MyMAAT). This study included 346 older adults aged 60 years and above with two or more chronic conditions (n = 346). Hypertension (30.2%), hyperlipidemia (24.0%), and diabetes (18.0%) were the most reported chronic conditions among participants. The mean score of treatment burden was 53.4 (SD = 28.2), indicating an acceptable burden of treatment. The mean score of health literacy was 16.4 (SD = 12.6), indicating a limited health literacy level among participants; meanwhile, the mean score of medication adherence was 32.6 (SD = 12.3), indicating medication non-adherence among participants. Medication adherence was significantly correlated with treatment burden (r = −0.22, *p* < 0.0001), health literacy (r = 0.36, *p* < 0.0001), number of chronic conditions (r = −0.23, *p* < 0.0001), and age (r = −0.11, *p* < 0.05). The study findings emphasize that multimorbid older adults with high treatment burdens and low health literacy are more likely to have poor medication adherence. This underscores the importance for clinicians to address these factors in order to improve medication adherence among older adults with multiple chronic conditions (MCC).

## 1. Introduction

The rise in life expectancy has shifted the focus of medical research to older people. As the number of chronic diseases rises with age, so does the number of treatments and prescribed medications. In Malaysia, the prevalence and factors associated with MCC in the older adult population in Malaysia have been well-established by Ghazali et al., using a large nationally representative sample of 3966 adults aged 60 years and above [1]. As the life expectancy in the older adult population increases, more older adults will spend longer portions of their lives with at least one or more chronic conditions associated with a disability or partial loss of bodily functions [2], multiple clinic appointments, frequent hospital visits, multiple medications, complex self-management routines, increased healthcare cost [3,4,5], poor quality of life [2], and cognitive decline and mortality [6], all of which may cause high treatment burden.

The term treatment burden refers to the “impact of the work of being a patient on functioning and well-being” [7]. The idea of treatment burden stemmed from healthcare professionals recognizing the shift in disease epidemiology from episodes of acute infectious diseases to non-communicable long-term chronic conditions such as diabetes or heart diseases, partial loss of bodily function, or life-limiting events such as stroke, Parkinson’s disease, or cancer. The new demands placed on patients to self-manage these conditions and the need for effective healthcare utilization to mitigate the experience of treatment burden. May et al. further implied that if the treatment burden is well understood and strategies to shift the burden materialize, this would carve a way for better health outcomes, improved adherence to treatment, and reduced healthcare cost [8]. Treatment burden arises when the responsibilities of self-managing older adults’ treatment activities outweigh their ability to handle them [9]. As the aging process takes place with deteriorating physical and cognitive abilities, older adults may become increasingly dependent on their family members or caretakers to manage their daily routines [10]. Therefore, multimorbid older adults have been associated with a greater risk of high treatment burden [11].

Most of the previous literature on treatment burden has been limited to single disease-specific burden; for instance, asthma [12,13], diabetes mellitus [14,15,16], cardiovascular diseases [13,17], and cancer [18,19]. Even though the idea of minimally disruptive medicine was first discussed in scientific studies almost a decade ago, the treatment burden from the perspective of a patient and the capacity to support patients with multiple morbidities has not yet been extensively scrutinized in practice [20]. Moreover, generalization of the study outcomes from these quantitative studies on the treatment burden of a specific condition in distinct geographical settings is challenging as the measure of treatment burden in individuals with similar conditions could vary depending on their education, social or family structure, income, support system, and care delivery system [7].

Older adults with MCC have been reported to also have low health literacy [21]. Health literacy is described as ‘the capacity of individuals to receive, process, and comprehend basic health information and services required to make wise health choices’, a description well-recognized by the U.S. Institute of Medicine and Healthy People, 2010 [22,23]. In other words, health literacy is a concept that concerns whether an individual is competent in gathering and using the information to maintain and improve their health. Older adults with multiple chronic conditions (MCC) often face difficulties in either accessing health information or receiving inadequate or conflicting health information from their care providers [21,24]. In a review article by McGilton et al. on the social and healthcare needs of multimorbid elderly, the authors reported that older adults with multimorbidity expect healthcare professionals to use fewer technical terminology and jargon and provide detailed explanations of diagnoses and the available treatment options, justification for prescribing particular medications to them without which they felt they have inadequate information for self-management of their chronic condition [21]. Additionally, the burden associated with their treatment plans and poor health literacy has been associated with challenges older adults face with adhering to their medications [7,25].

Non-adherence to medication represents a socially fundamental dilemma, especially when associated with the older adult population coping with MCC. According to WHO, medication adherence is described as the level to which an individual’s behavior conforms to a healthcare provider’s recommendation [26]. As older adults face a higher risk of developing MCC, which may require them to be on numerous medications, they may become predisposed to the risk of polypharmacy, frailty, and, consequently, a higher risk of non-adherence to medication [27]. The complexity of their treatment regime further potentiates non-adherence to treatment plans and medication in the elderly population. Non-adherence to medication is associated with worse health outcomes, disease progression, increased hospitalization, treatment failure, and increased expenditure on healthcare [28]. Barriers to medication non-adherence among the elderly include treatment burden, inadequate health literacy, multiple comorbidities, multiple care providers, communication barriers, and complex drug regimens [11,29,30]. Safran et al., in their study, explained the relationship between treatment burden and medication adherence. They estimated 25% of their elderly patients took lesser medications than prescribed in order to avoid treatment burden, which is directly linked to their financials [31]. Health literacy skills (e.g., reading labels, numeracy, reading insulin pen dose scales) are often required in taking medications for chronic diseases such as diabetes mellitus and cardiovascular disease and may be a complex task for older adults. Davis et al. reported that in a group of older primary care adults, only 30% of the patients were able to articulate the instruction on their medication container labels, suggesting a lack of comprehension skills [32].

Understanding and recognizing treatment burden and health literacy levels among multimorbid elderly could lead to reduced hospitalizations, better disease management, or improved quality of life; it serves as a relevant health quality indicator for healthcare providers treating geriatric patients. To date, there is a dearth of quantitative studies conducted globally that have investigated the impact of treatment burden and health literacy on medication non-adherence among older adults with MCC, limiting our understanding of this critical area. To the best of our knowledge, this study represents the first attempt in Malaysia to examine the relationship between treatment burden, health literacy, and medication non-adherence among older adults with MCC, filling a significant research gap in the local context. The primary objectives of this study were to assess the impact of treatment burden and health literacy on medication adherence among older adults with MCC and to explore the potential moderating effects of demographic and clinical factors.

## 2. Materials and Methods

### 2.1. Study Design and Population

A prospective, cross-sectional study was carried out to examine the relationship between treatment burden and health literacy with medication adherence in older adults coping with MCC in the state of Selangor, Malaysia. The study was performed using a convenience sample of the elderly population aged ≥60 years. Ten general practitioners (GP) clinics in Selangor were conveniently approached and selected for inclusion in the study. The participants aged 60 years and above, had a diagnosis of two or more chronic conditions, able to understand and respond to the interview in English or Malay were included in this study. Informed consent was obtained from every participant prior to enrolling in this study. The sample size was determined based on previous data by Ghazali et al., that the prevalence of older adults suffering from MCC is 40.6% [9]. It was calculated by utilizing the Raosoft^®^ sample size calculator with a population size of 3.5 million people over the age of 60, a margin error of 5% with a confidence interval of 95% [33]. The computed minimum sample size was 371.

A comprehensive, validated, structured interview questionnaire was used to collect the patients’ data. A pilot study was carried out with 30 patients to ensure internal consistency and face validity of the questionnaire, the time needed to answer the questions, and to identify any concerns that may arise during data collection. Good consistency and reliability were achieved for the questionnaire; using the coefficient alpha, Cronbach’s Alpha, for which the value obtained was 0.82. The interview questionnaire included (a) demographic data: age, gender, ethnicity, level of education, employment status, household income, and the number and type of chronic conditions suffered using the list of 46 chronic conditions by the WHO’s 10th revision of the International Statistical Classification of Diseases and Related Health Problems, (b) treatment burden assessed using the Burden of Treatment Questionnaire (TBQ-15), (c) health literacy of participants assessed using the Short Form Health Literacy Questionnaire (HLS-SF12), and (d) medication adherence level of participants assessed using the Malaysia Medication Adherence Assessment Tool (MyMAAT).

### 2.2. Treatment Burden Instrument

The TBQ-15 was originally developed in 2011 to objectively assess the treatment burden of chronic diseases for patients suffering from MCC and showed a Cronbach’s alpha of 0.90 and retest reliability of 0.77 [7]. The questionnaire comprised 15 questions that assessed the burden associated with medication administration, care provider visits, laboratory tests, self-monitoring activities, organizational tasks, physical activity and diet, and the social burden of coping with chronic conditions without limiting its questions to a single chronic condition, scored on a Likert scale between scores 0 to 10 [34]. Scores are added together to obtain a cumulative score between 0 to 150, with larger score numbers representing greater degrees of treatment burden. The TBQ-15 questionnaire follows five domains: 2-item on lifestyle change burden linked with lifestyle changes, e.g., nutrition, physical activity, and sleep; 2-item on social life burden related to social life, e.g., relationships; 5-item on medication burden related to medication usage and experienced side effects; 5-item on time and the administrative burden related to self-managing, medical appointments, administrative duties and 1-item on financial burden [4]. The Patient Acceptable Symptom State (PASS) for the burden of treatment was a TBQ score of 59/150 [35], above which treatment burden was denoted as high and below which treatment burden was denoted as acceptable for participants in the present study.

### 2.3. Health Literacy Instrument

The health literacy of participants was examined using the HLS-SF12, a shorter adaptation of the 47-question Health Literacy European Questionnaire (HLS-EU-Q47), validated in six Asian nations, including Malaysia. The HLS-SF12 model showed good validity and reliability with a Cronbach’s alpha value of 0.85. They assessed four distinct dimensions of health literacy; “access, understanding, appraisal, and application of health information in 3 areas: health promotion, disease prevention, and healthcare” scored using a four-point Likert-scale ranging from score 1 (very easy) to score 4 (very difficult) without repeating any items [36]. All scores were cumulated to a minimum of 0 and maximum of 50, which was then stratified into 4 levels of health literacy: limited health literacy level (score 0–24), problematic (score 25–33), sufficient health literacy level (score 34–42), and excellent health literacy level (score 43–50) in this present study.

### 2.4. Medication Adherence Instrument

The medication adherence of the study participants was assessed using the validated 12-item MyMAAT. The MyMAAT scale was reliable, with excellent internal reliability and Cronbach’s alpha value of 0.91 [37]. MyMAAT comprised 21 questions which were then reduced further to 12 final questions revolving around 5 themes: “patients’ medication-taking behaviour, perceived utility of the medications–benefits, costs, and efficacy, perceived barriers to medication adherence, perceived self-efficacy and social support and perceived severity and susceptibility of diabetes” [37]. The adherence score in the present study was calculated using the 5-point Likert scale ranging from score 1 (strongly agree) to score 5 (strongly disagree), with the total score ranging between a minimum of 12 to a maximum of 60. The level of medication adherence was stratified into 2 levels according to the cumulative score: adherent (scores 54–60) and non-adherent (scores 12 to 53).

### 2.5. Data Collection and Analysis

Data collection was conducted through a structured face-to-face interview by the researcher, and it took around 10–15 min to interview every older adult. The face-to-face interview was selected as the older adults may respond only when they were involved in a direct conversation with the researcher. Verbal responses of the respondents were recorded by the interviewer in the assessment form; random code numbers were allocated to the participants to maintain anonymity. The collected data were tabulated and analyzed by using Statistical Package for Social Sciences (SPSS) version 26.0. Descriptive statistics (i.e., frequencies, percentages, mean (X), and standard deviation (SD) were used to summarize the demographic characteristics of the participants. In order to present the qualitative data, frequency tables, numbers, and percentages were used and examined using Chi-square (χ^2^) test. Pearson correlation coefficient test (r) was utilized to explore the correlation between the level of medication adherence, treatment burden, health literacy, and demographic variables among the elderly with multiple comorbidities. A *p*-value <0.05 was considered statistically significant in the present study.

### 2.6. Ethical Approval

Approval for the present study was obtained from the International Medical University Joint Committee on Research and Ethics (IMU-JC) (Approval number: MPP 1-2022(04)).

## 3. Results

### 3.1. Demographic Characteristics of Participants

The preliminary data collected for this study included 346 older adults aged 60 years and above with two or more chronic conditions (n = 346). During data collection, 371 potential participants were approached to participate in the study; however, 25 declined to participate, and finally, a total of 346 older adults participated in this study. The response rate was found to be 93%. The demographic variables for the study sample included: age, gender, race, education, employment status, and household income. The mean age of the participants was 67.1 years, ranging from 60 to 89 years old. The majority of the sample was female (52.3%). Most participants were Malay (64.5%), had pursued their education up to secondary level (45.1%), and had retired (58.7%). Most participants had a household income of less than MYR 2000 (23.7%), between MYR 3001 to MYR 4000 (27.7%), and between MYR 4001 and MYR 5000 (22.8%). Participants’ demo-graphic characteristics are shown in Table 1.

**Figure 1:**
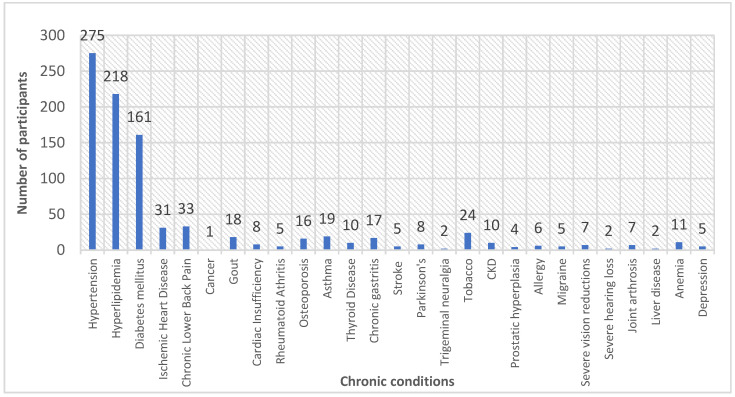
Distribution of chronic conditions among the participants (n = 346).

The mean number of chronic conditions among participants was 3. Hypertension (30.2%), hyperlipidemia (24.0%), and diabetes (18.0%) were the most reported chronic conditions among participants, followed by chronic lower back pain (3.6%), ischemic heart disease (3.4%) and tobacco use (2.6%). The details are presented in Figure 1. The number of chronic conditions was significantly associated with increasing age (*p* < 0.05). The details are presented in Table 2. The mean number of chronic conditions was statistically significantly higher among females (3.19 ± 0.89) than males (2.85 ± 0.78) (*p* < 0.05). Regarding the educational level, there was a significant decrease in the mean number of chronic diseases with increasing educational level; up to diploma level (*p* < 0.05) and a significant increase in the mean number of chronic diseases at the university level (3.03 ± 0.83) (*p* < 0.05).

### 3.2. Assessment of Treatment Burden among the Participants

**Figure 2:**
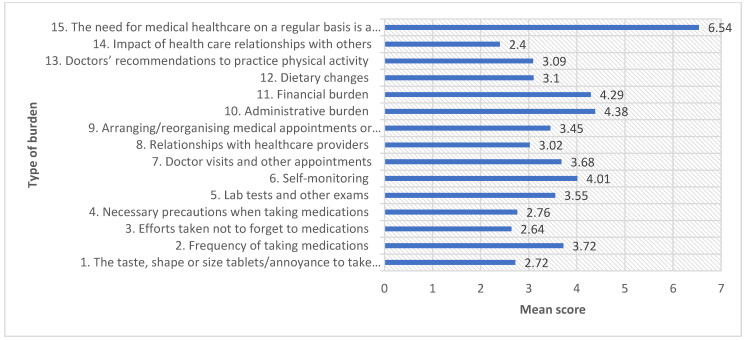
The mean score of participants’ responses to the TBQ-15 questionnaire (n = 346).

The TBQ-15 questionnaire had a possible score of 0–150. The mean score of the treatment burden for the participants was 53.4 (SD = 28.2), indicating an acceptable burden of treatment according to the Patient’s Acceptable Symptom State (PASS). Around 47.6% of participants scored a total score level above 59, denoting a high treatment burden level. Based on the TBQ-15 scores, the highest mean scores were obtained for aspects such as the need for medical healthcare on a regular basis as a reminder of health problems suffered (Q15) with a mean score of 6.54, administrative burden (Q10) with a mean score of 4.38 and a financial burden (Q11) with a mean score of 4.29. The details are presented in Figure 2. Meanwhile, the lowest mean scores were obtained for aspects such as the impact of healthcare relationships with others (Q14), with a mean score of 2.40, efforts taken not to forget the medication (Q3), with a mean score of 2.64 and the taste, shape, or size of tablets annoyance to take medication (Q1) with a mean score of 2.72. Around 58.4% of participants reported a very high treatment burden for Q15. When each of the domains of treatment burden was analyzed individually, the domains with the greatest level of burden were the social life burden (mean = 4.47, SD = 2.29), financial burden (mean = 4.29, SD = 3.26), and time and administrative and time burden (mean = 3.71, SD = 2.37).

### 3.3. Assessment of Health Literacy among the Participants

**Figure 3:**
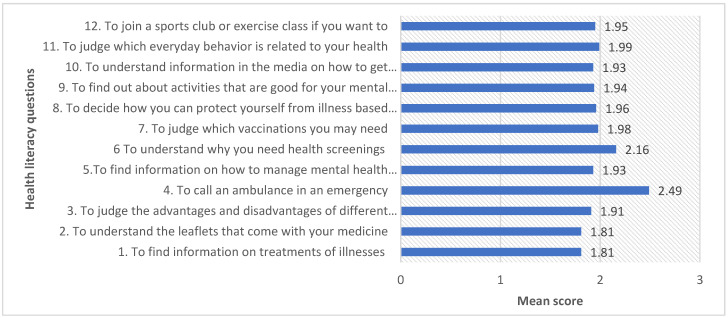
The mean score of participants’ responses to the HLS-SF12 questionnaire (n = 346).

The HLS-SF12 questionnaire has a possible score of 0–60. The mean score of health literacy for the participants was 16.4 (SD = 12.6), indicating a limited health literacy level among participants. About three fourth of participants reported limited health literacy; meanwhile, only 2% of participants reported an excellent health literacy level. The mean score of participants’ responses to the HLS-SF12 questions, which ranged from 1 (very difficult) to 4 (very easy), is presented in Figure 3. Most participants responded that they experienced great difficulty (score 1) in finding information on treatments for illness (Q1), understanding leaflets that come with their medication (Q2), finding out about activities that are good for their mental well-being (Q9), and deciding how they can protect themselves from illness based on advice (Q8). When comparing health literacy based on the three areas of health literacy, healthcare, disease prevention, and health promotion, there was not much disparity observed between overall mean scores and gender-specific mean scores of each area of health literacy.

### 3.4. Assessment of Medication Adherence among the Participants

**Figure 4:**
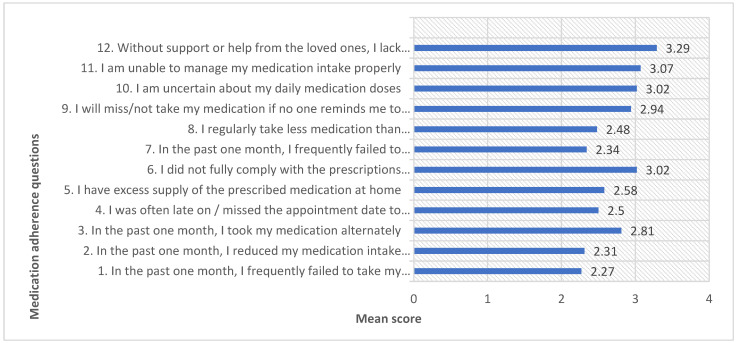
The mean score of participants’ responses to MyMAAT questionnaire (n = 346).

The MyMAAT questionnaire had a possible score between 12 to 60. The mean score of medication adherence for the participants was 32.6 (SD = 12.3), indicating medication non-adherence among participants. Of the total, 83.2% of participants were non-adherent to their medications. Most participants reported difficulty in aspects such as Q1, adhering to medications according to their doctor’s instructions (61.0%); Q2, reducing their medication intake whenever they felt better (57.8%); Q7, frequently failing to remember to take their medications (59.3%); and Q8, regularly taking fewer medications than prescribed due to fear of side effects (53.8%). The data are presented in Figure 4.

### 3.5. Correlation of Treatment Burden and Medication Adherence Level

Table 3 reveals that more than half of the participants with acceptable treatment burden levels (88.9%) and high treatment burden levels (98.5%) were non-adherent to their medications (*p* < 0.05). Treatment burden was significantly negatively correlated to medication adherence.

### 3.6. Correlation of Health Literacy and Medication Adherence Level

More than half the participants from all levels of health literacy, limited (95.0%), problematic (90.2%), sufficient (83.3%), and excellent (66.7%), were all non-adherent to their medications (*p* < 0.05). Health literacy was significantly positively correlated to medication adherence. The details are presented in Table 4.

### 3.7. Correlation between Medication Adherence, Treatment Burden, Health Literacy

Pearson’s correlation coefficient matrix between treatment burden, health literacy, number of chronic conditions, and age was performed. Both treatment burden and health literacy were associated with medication adherence. A significant negative correlation (r = −0.22, *p* < 0.0001) was found between treatment burden and medication adherence, indicating that lower treatment burden contributed to higher medication adherence levels. Meanwhile, a significant positive correlation (r = 0.36, *p* < 0.0001) was found between treatment burden and health literacy, indicating that high health literacy contributed to higher medication adherence levels. Health literacy was negatively correlated to the number of chronic conditions (r = −0.20, *p* < 0.001) and age (r = −0.20, *p* < 0.001). This illustrated that low health literacy was associated with a higher number of chronic conditions and increasing age. Medication adherence was also statistically significantly correlated negatively to the number of chronic conditions (r = −0.23, *p* < 0.001) and age (r = −0.11, *p* = 0.04), which showed that the lower medication adherence levels were associated with a greater number of chronic conditions and increasing age of participants. As the number of chronic conditions was statistically significantly correlated positively with age (r = 0.15, *p*= 0.005), the data showed that a greater number of chronic conditions was associated with increasing age.

## 4. Discussion

The results of the present study showed that most of the participants had a mean age of 67.1 years, were Malay women, and had pursued their education up to the secondary level. These results were consistent with Ghazali et al., whereby 66.5% of their study participants coping with multiple chronic conditions were aged between 60 to 69 years old, 51.1% were female, 57.6% were Malay, and 43.6% attended secondary school [1]. Hypertension, hyperlipidemia, and diabetes were the most reported chronic conditions among participants, paralleled by reports of disease burden and global death rate caused by cardiovascular diseases and diabetes mellitus [38,39,40].

The mean number of chronic conditions was statistically significant among females compared to males. Other past studies have also revealed that females are more likely to have a higher number of chronic conditions in comparison to males [41,42]. In the present study, despite the country having a gender ratio of 106 males for every 100 females in 2021 [43], the dominance of female participants could also be explained by their health-seeking behavior. In a study by Mohd Noh, the author reported study outcomes from the National Health and Morbidity Survey (2019), whereby the female gender was more prevalent in reporting sickness [44]. The present study showed a significant association between the mean number of chronic conditions and age. The association was advocated by similar studies revealing that the number of chronic conditions increased with age [42,45]. In a cross-sectional study of over 1.7 million Scottish people, the authors reported incremental mean number of morbidities with age; mean of 1.18 in those aged between 45 to 64 years old, 2.60 in those aged between 65 to 84 years old, and 3.62 in those aged 85 years and above (*p* < 0.0001) [46]. In addition to the global increase in longevity of life expectancy explaining the present results, Fabbri et al. set forth an interesting reason for the observed results, whereby a steeper increment in an inflammatory marker, interleukin-6 predicted an augmented increase in the number of chronic conditions with increasing age [47]. With regard to education, a significant negative association exists between education and the mean number of chronic conditions. Past studies have reported a higher prevalence of chronic conditions in those with no or low education levels [45,48,49]. A higher prevalence of chronic conditions seen across lower educated groups may be explained by multiple unmeasurable factors such as diet, psychosocial factors, unemployment, stress, and access to health services [49,50].

The study reported a mean score of 53.4 for the treatment burden experienced by the study participants. In the original French version of the TBQ-13 questionnaire, Tran et al. stratified treatment burden scores into three distinct, non-adjusted clusters based on global score cut-off points; low treatment burden with a global score of 11.3 (SD = 9.2), moderate treatment burden with a global score of 34.6 (SD = 11.1) and high treatment burden with a global score of 65.8 (SD = 18.1) [35]. If similar cut-off points were compared with the mean score obtained from the present study, the reported global mean score in the study would be in the moderate treatment burden cluster. Meanwhile, Sav et al. noted a comparable mean global treatment bur-den score of 56.5 (SD = 34.5) from a total score of 150 using the TBQ-15 questionnaire [4]. The present study reported that three-fifths of participants reported a high treatment burden. This is comparable with results by El-Nagar et al. in Egypt, whereby 50% of their study participants reported a high treatment burden [51]. In a similar study in 2022, a population-based Danish evaluation of treatment burden showed only 13% had a high treatment burden; meanwhile, about 69% had little or no treatment burden [52]. The differences in the treatment burden score observed across these studies can be attributed to socio-demographic differences in where each of the studies took place, differences in treatment of multimorbidity by physicians, and differences in the questionnaire used to measure treatment burden in these studies. Another reason for this disparity is due to the treatment burden being a dynamic and multidimensional process. Sav et al. illustrated that over time, individuals could reach a state of disease acceptance or treatment familiarity [3]. This process could lessen the treatment burden on these individuals.

The present study reported that 75% of participants had limited health literacy levels, and when a comparison was made between their gender counterparts, no differences were seen. In a study by Yunus et al., studying health literacy levels among elderly Malaysian aged 60 years and above, the authors reported that 27.7% of participants had limited health literacy levels, 35% had problematic literacy levels, 25.2% had sufficient health literacy levels, and the remaining 12.1% exhibited excellent literacy levels [53]. Similar to the present study, no differences were observed across the three domains of health literacy; however, the male population was observed to have a higher health literacy level compared to females. Seeing as the male counterpart in the present study were more exposed to education than females, they most presumably had more encounters with the healthcare system, giving them more chances to develop their health literacy levels. Although this was a noteworthy finding, contradicting results have been reported on the effect of gender on health literacy levels [54,55,56]. Future research could look into potential explanations for possible gender differences in reported health literacy levels. Past studies reported comparably similar results, whereby more than half of the participants reported limited health literacy levels [55,57]. However, disparities in the proportion of health literacy levels reported in other previous studies [56,58] can be explained by differences in age, health status, socio-economic and cultural backgrounds of participants, and the health literacy measure tools. For example, Smith et al. reported disparities in the proportion of participants who had limited or marginal health literacy levels when using different health literacy tools; 48% using the Newest Vital Sign (NVS) tool, 26% using the Test of Functional Health Literacy in Adults (TOFHLA) tool and 22% using The Rapid Estimate of Adult Literacy in Medicine (REALM) tool [55].

The results of the present study also reported that about more than 80% of the participants were non-adherent to their medications. In a study by Neoh et al. of the multi-ethnic geriatric cohort, the authors reported non-adherence among 49.4% of their participants [59]. Meanwhile, a randomized controlled trial examining the effects of doctor-pharmacist collaboration initiatives on health-related outcomes among elderly Malaysians aged 65 years and above and taking at least five medications reported 65.8% of participants were non-adherent to their medications [60]. The present study showed a majority of non-adherent patients were forgetful, feared the experience of medication side effects, and intentionally skipped medications when they felt better. Similar factors of non-adherence to medications were reported in other studies, with forgetfulness being an unintentional factor while most others being intentional factors for medication non-adherence [60,61,62,63]. A high proportion of non-adherence seen in the present study can be seamlessly multifactorial and be elucidated by the cultural background and health literacy level of the participants, accessibility to healthcare facilities, levels of delinquency suffered [63], age-related pharmacokinetic and pharmacodynamic changes [64] and the number of chronic conditions suffered successively leading to polypharmacy. Nevertheless, the effect of some of these factors was not able to be tested in the present study.

In regard to the correlation between treatment burden and medication adherence, the present study showed a significant negative correlation. The threshold of medication adherence decreases with increasing treatment burden. Rogers et al., and Eton et al., illustrated a significant association between treatment burden and medication adherence, specifically relating to the burden of medical information and medication [11,65]. Schreiner et al. also reported a significant negative correlation between both cumulative and task-specific treatment burden and medication adherence (*p* < 0.001) [66]. The experience of burden suffered by these individuals seamlessly was related to the number of medications taken, side effects and drug–drug interactions, concerns about developing dependence, frustration on how taking medications interferes with some of their daily routines, and changes in medication packaging or brand all of which reduces medication adherence [67,68]. Often, non-adherence to medication regimes becomes more apparent when individuals suffer from medication-related treatment burdens. In a study of geriatric patients above 65 years old in Kuwait, approximately three-fifths of participants heed their concerns regarding side effects seriously (58.5%; 95% CI: 53.6 to 63.2) and are worried about forgetting to take their medications (69.1%; 95% CI: 64.4 to 73.4); more than half of individuals (46.3%; 95% CI: 41.4 to 51.1) strongly agreed that side effects had a negative impact on their wellbeing, were unpleasant (42.5%; 95% CI: 37.7–47.3), and interfered with their daily lives (42.2%; 95% CI: 37.5–47.1); more than half of the participants reported that their lives were primarily focused around taking their medications (95% CI: 52.7–62.3 and 33.3% reported disruption with everyday work, social interactions, or leisure activities (95% CI: 23.7–32.4) [69]. Perhaps, the most noteworthy observations are medication non-adherence worsens health outcomes in older adults, and identification of these burdens related to medication adherence by clinicians could facilitate and inform clinicians on potential solutions to improving medication adherence.

A significant positive correlation between health literacy and medication adherence was also observed in the present study. This is not unusual, as research shows that managing medication regimens in older adult populations can be difficult [70]. Since examining the relationship between health literacy and medication adherence in a multimorbid older population is still relatively new, few research works are available that are comparable with the present study. The current findings are best contrasted with recent studies that have looked into the relationship between health literacy and medication adherence regardless of health status. Aranha et al., in their cross-sectional study of older African American adults aged 60 years and above, noted a significant correlation between health literacy and medication adherence (*p* = 0.001) [71]. Similar positive associations were reported by multiple other studies [72,73,74]. Mayo-Gamble and Mouton reported that individuals with adequate health literacy (60.8%) were more likely to remember to take their medications than those with inadequate health literacy [73]. While this may be the case, some past studies have reported contradicting results showing no association between health literacy and medication adherence. Of noteworthy, in a systematic review by Schonfeld et al., the authors put forward that the conflicting results reported by these past studies may be due to the inclusion of a broad selection of validated tools of health literacy and improper inclusions of unreported younger adults and subgroups, which may obscure the true association between health literacy and medication adherence among older adults [75]. Secondly, intentional and unintentional non-adherence among these older adults may partly muddle this association. Older adults with inadequate or marginal health literacy levels were more likely to unintentionally non-adhere to their medication discharge orders, while those with adequate health literacy levels were more likely to deliberately act out similarly [76].

The present study also showed a significant correlation between the number of chronic conditions and its relationship with medication adherence and treatment burden. However, past studies examining a correlation between the number of chronic conditions and medication adherence were conflicting [76,77,78,79]. These may potentially be due to the inclusion of a broad age subgroup of participants in these past studies or how the study participants were sampled in the different studies, obscuring the true effect of the association between the number of chronic conditions and medication adherence among older adults. The association between the number of chronic conditions and treatment burden was supported by Morris et al. [80]. Meanwhile, Hounkpatin et al. noted that an increase in treatment burden levels in their older aged participants was associated with having greater than five chronic conditions [81].

In terms of health literacy and its correlation with age and the number of chronic conditions, the current study found a significant negative relationship between health literacy and the number of chronic conditions and age. Likewise, Rheault et al., in a cross-sectional study of indigenous Australians with chronic diseases noted that low levels of health literacy were associated with ages above 55 years old and having more than one chronic condition [82]. Wolf et al.’s and Patel et al.’s studies also supported the correlation between health literacy and age. Health literacy involves a number of cognitive functions, including comprehension, problem-solving, making comparisons, reasoning, and computation [83]. With increasing age, older adults may experience a decline in their memory and cognitive power that limit their health literacy [83,84].

### Limitations

Nevertheless, there are some limitations to the present study. The sample collected in the study was a convenience sample of ten clinics in the Selangor region, potentially introducing some selection bias that might hinder generalization. To minimize this, convenience sampling was supplemented with a random sampling of participants from each clinic. The findings of the study may also differ according to the localities of the study setting, such as semi-urban and rural areas. As the study included patients visiting GP clinics, patients with MCC who cannot attend GP clinics or had follow-ups in tertiary hospital clinics, or very sick patients with MCC who are hospitalized in tertiary hospitals, nursing facilities, etc., were unintentionally excluded. In addition to that, the responses for the study were captured through face-to-face interviews, which may have introduced social desirability bias where participants might have given favorable answers to fit into the notion of a ‘good patient.’ To minimize this, the interview was conducted by the researcher or clinician in a neutral manner using a structured interview guide containing demographic questions and study instruments. The study’s findings should be interpreted with consideration due to the study being cross-sectional. It is not possible to establish causation or the directionality of the associations observed. Despite the limitations, the current findings of the study provide imperative information on the treatment burden, health literacy, and medication adherence level of older adults coping with MCC in Malaysia.

Although several studies have previously assessed treatment burden, health literacy, and medication adherence independently in older adults with MCC, to the best of our knowledge, this was the first study in Malaysia to assess the association of treatment burden and health literacy with medication adherence in this target population, thus highlighting the need of recognizing avoidable treatment burden and factors affecting health literacy. As treatment burden and health literacy are correlated with adherence to medication, measuring treatment burden and health literacy as potential health quality indicators may provide important information for healthcare workers in developing strategies to help patients to attain optimal adherence levels.

Nevertheless, further research is crucial to study the antecedents of treatment burden and health literacy and its impact on medication adherence in wider regions of the country. Prevailing future studies to explore interventions to reduce treatment burden and improve health literacy and medication adherence in the target population would also be seamlessly valuable.

## 5. Conclusions

The present study demonstrated that almost three-fifths of participants suffered from a high treatment burden, three-fourths of participants had limited health literacy levels, and about four-fifths of participants were non-adherent to their medications. Although several studies have previously assessed treatment burden, health literacy, and medication adherence independently in older adults with MCC, to the best of our knowledge, this was the first study in Malaysia to assess the association of treatment burden and health literacy with medication adherence in this target population, thus highlighting the need of recognizing avoidable treatment burden and factors affecting health literacy. As treatment burden and health literacy are correlated with adherence to medication, the adaptation of treatment burden, health literacy, and medication adherence instruments into clinical practice when treating older adults with MCC as potential health quality indicators may provide important information for healthcare workers in developing strategies to help patients attain optimal medication adherence levels and provide self-management support.

## Figures and Tables

**Table 1:** Demographic details of the participants (n = 346).

Variables	Range	Mean	SD
Age (years)	60–69	67.1	6.3
Number of chronic conditions	3–6	3	
**Variables**	n (%)		
**Sex**			
Male	165 (47.7)		
Female	181 (52.3)		
**Race**			
Malay	223 (64.5)		
Chinese	35 (10.1)		
Indian	88 (25.4)		
Others	0		
**Education**			
Never been to school	34 (9.8)		
Primary	97 (28.1)		
Secondary	156 (45.1)		
Diploma	44 (12.7)		
University	15 (4.3)		
**Employment status**			
Employed	47 (13.6)		
Unemployed	96 (27.7)		
Retired	203 (58.7)		
**Household income**			
<MYR 2000	82 (23.7)		
MYR 2001–MYR 3000	63 (18.2)		
MYR 3001–MYR 4000	96 (27.7)		
MYR 4001–MYR 5000	79 (22.8)		
MYR 5001–MYR 10,000	26 (7.6)		
>MYR 10,000	0		

**Table 2:** Demographic characteristics and the mean number of chronic conditions (n = 346).

Variables	Number n (%)	Number of Chronic Conditions (Mean ± SD)	*p*-Value
**Age (years)** 60–69 70–79 80–90	234 (67.6) 91 (26.3) 21 (6.1)	2.93 ± 0.83 3.26 ± 0.89 3.05 ± 0.80	*p* = 0.007
**Sex**			
Male	165 (47.7)	2.85 ± 0.78	*p* = 0.000
Female	181 (52.3)	3.19 ± 0.89	
**Race**			
Malay	223 (64.5)	3.10 ± 0.91	
Chinese	35 (10.1)	2.86 ± 0.65	*p* = 0.100
Indian	88 (25.4)	2.91 ± 0.78	
Others	0	0	
**Education**			
Never been to school	34 (9.8)	3.50 ± 0.86	
Primary	97 (28.1)	3.02 ± 0.90	
Secondary	156 (45.1)	2.98 ± 0.82	*p* = 0.006
Diploma	44 (12.7)	2.80 ± 0.79	
University	15 (4.3)	3.03 ± 0.83	
**Employment status**			
Employed	47 (13.6)	2.60 ± 0.80	
Unemployed	96 (27.7)	3.12 ± 0.93	*p* = 0.001
Retired	203 (58.7)	3.06 ± 0.81	
**Household income**			
<MYR 2000	82 (23.7)	3.00 ± 0.92	
MYR 2001–MYR 3000	63 (18.2)	3.00 ± 0.84	
MYR 3001–MYR 4000	96 (27.7)	3.25 ± 0.93	*p* = 0.010
MYR 4001–MYR 5000	79 (22.8)	2.92 ± 0.71	
MYR 5001–MYR 10,000	26 (7.6)	2.65 ± 0.63	
>MYR 10,000	0	0	

SD: Standard deviation.

**Table 3:** Correlation of medication adherence level by treatment burden levels.

Treatment Burden Level	Medication Adherence Level		X^2^; *p*-Value
	Adherent	Non-Adherent	
Acceptable treatment burden	24 (11.2%)	190 (88.8%)	X^2^ = 11.1; *p* = 0.001
High treatment burden	2 (1.5%)	130 (98.5%)	

**Table 4:** Correlation of medication adherence level by health literacy levels.

Health Literacy Level	Medication Adherence Level		X^2^; *p*-Value
	Adherent	Non-Adherent	
Limited	13 (5.0%)	245 (95.0%)	X^2^ = 14.0; *p* = 0.003
Problematic	6 (9.8%)	55 (90.2%)	
Sufficient	3 (16.7%)	15 (83.3%)	
Excellent	3 (33.3%)	6 (66.7%)	

## Data Availability

The data presented in this study are available on request from the corresponding author. The data are not publicly available due to ethical restrictions.
